# Redefining the Axillary Aesthetic: Surgical Management of Axillary Tissue Hypertrophy

**DOI:** 10.3390/medicina60010126

**Published:** 2024-01-10

**Authors:** Neil Tanna, Sarah Barnett, Christopher Aiello, Lucas M. Boehm, M. Bradley Calobrace

**Affiliations:** 1 Division of Plastic and Reconstructive Surgery, Northwell Health, Great Neck, NY 11021, USA; sarah.barnett@pennmedicine.upenn.edu (S.B.); caiello@northwell.edu (C.A.); 2 Donald & Barbara Zucker School of Medicine at Hofstra/Northwell, Hempstead, NY 11549, USA; 3 CaloAesthetic Plastic Surgery, Division of Plastic Surgery, University of Louisville, Louisville, KY 40222, USA; drboehm@parkinsplasticsurgery.com (L.M.B.); drbrad@calobrace.com (M.B.C.)

**Keywords:** breast surgery, axillary tissue, aesthetic surgery

## Abstract

*Background and Objectives:* Axillary tissue hypertrophy consists of ectopic breast tissue and occurs in up to six percent of women. Women complain of pain, interference with activity, and dissatisfaction with appearance. While it is recommended that accessory breast tissue be removed via surgical excision, there is lack of consensus on the best technique for the surgical management of axillary tissue hypertrophy. In this study, the senior authors (BC and NT) review outcomes and complications as they pertain to the surgical treatment of axillary tissue hypertrophy and axillary contouring. *Materials and Methods:* A retrospective review of all patients (*n* = 35), from two separate institutions, who presented with axillary tissue hypertrophy between December 2019 and August 2021 was conducted. All patients underwent a technique that included direct crescentic dermato-lipectomy and glandular excision with axillary crease obliteration. Tissue was sent for histological analysis after removal. During a six-month follow-up period, all patient outcomes were recorded. *Results:* The authors treated 35 women with axillary tissue hypertrophy. All patients complained of aesthetic deformity with significant discomfort leading to the desire for surgery. Histologically, all specimens contained benign breast and adipose tissue. Hypertrophic scarring, seroma, and axillary cording were noted complications. *Conclusions:* Detailed is the surgical management and optimal technique that can be used to treat both adipose and fibroglandular axillary tissue hypertrophy while simultaneously providing a favorable axillary aesthetic.

## 1. Introduction

**Figure 1:**
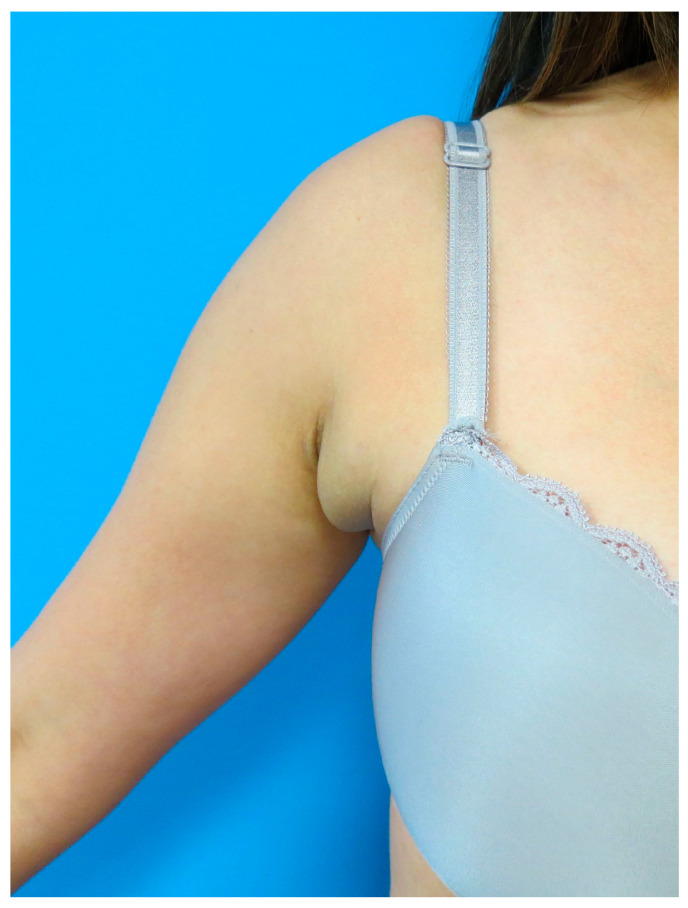
**Axillary tissue hypertrophy.** Axillary ectopic breast tissue consists of ectopic mammary tissue that develops outside the normal breast region. Most commonly it is found in the axilla but can occur anywhere along the embryonic mammary streak.

Axillary ectopic breast tissue consists of ectopic mammary tissue that develops outside the normal breast region. Most commonly, it is found in the axilla but can occur anywhere along the embryonic mammary streak (Figure 1). Accessory breast tissue originates from an embryonic mammary ridge which forms during the sixth week of development. The ridge is a bilateral thickening of the ectoderm that runs from the axillae to the inguinal region and rapidly regresses, except for in the thorax [1]. In the case of ectopic breast tissue, this embryonic mammary ridge, also referred to as the milk line, fails to degenerate. Most cases of axillary ectopic breast tissue are sporadic, but others can follow an autosomal dominant inheritance pattern [2].

Since axillary ectopic breast tissue is derived from normal breast tissue, it can contain all elements of normal breasts and tends to respond to cyclical hormonal changes. In fact, this condition is most often asymptomatic until puberty, after which ectopic tissue is hormonally stimulated. After pregnancy, axillary tissue can increase in size, become engorged and even secrete milk through pores of the skin leading to tenderness, irritation, and discomfort [3]. Furthermore, as with normal breast tissue, axillary ectopic breast tissue can acquire benign and malignant pathologies [4].

Up to 6% of women and 3% of men suffer from this anomaly that is often bilateral in nature [5]. Although the risk of malignancy is low, and some patients are asymptomatic, axillary ectopic breast tissue can cause significant distress. Patients complain of exacerbation of pain with hormonal surges, interference with daily activities, restriction of arm movement/posture, irritation from clothing, as well as increased anxiety and stress. In addition to functional impairment, patients commonly present with cosmetic concerns. For these reasons, surgical intervention is often recommended [6,7].

Historically, axillary ectopic breast tissue has received sparse attention in the literature. The ideal method of surgical treatment is yet to be established. Direct excision of the accessory breast tissue allows for complete resection but has been criticized as it can disrupt lymphatic channels and lead to the formation of dead space if not executed with the correct technique. Liposuction allows for the effective extraction of adipose tissue [8]; however, it is inadequate for the removal of fibroglandular breast tissue and does not address excess skin. For these reasons, some have suggested a possible combination of direct excision and liposuction when managing this condition [2]. Recent publications have introduced the use of microdebriders, mammotones, and ultrasound-guided liposuction devices as options for surgical care [8,9,10,11]. The current study presents a direct excision technique to create a smooth transition between the axilla and breast mound by fully excising excess skin and adipo-glandular ectopic axillary tissue and obliterating the axillary crease to achieve optimal aesthetic results.

## 2. Materials and Methods

All female patients who had undergone an excision of axillary tissue hypertrophy by two separate surgeons (BC and NT) were included. The study period was from December 2019 to August 2021. Deidentified information was collected on various patient demographic variables including sex, age, and body mass index. Outcome variables retrospectively assessed include length of follow-up, postoperative adverse events, complications, management of complications, and pathology reports. The sample size reflects the patient population available and was not statistically powered. All patients underwent the same technique of direct skin and adipo-glandular axillary tissue excision with axillary crease obliteration.

### 2.1. Preoperative Demarcation

**Figure 2:**
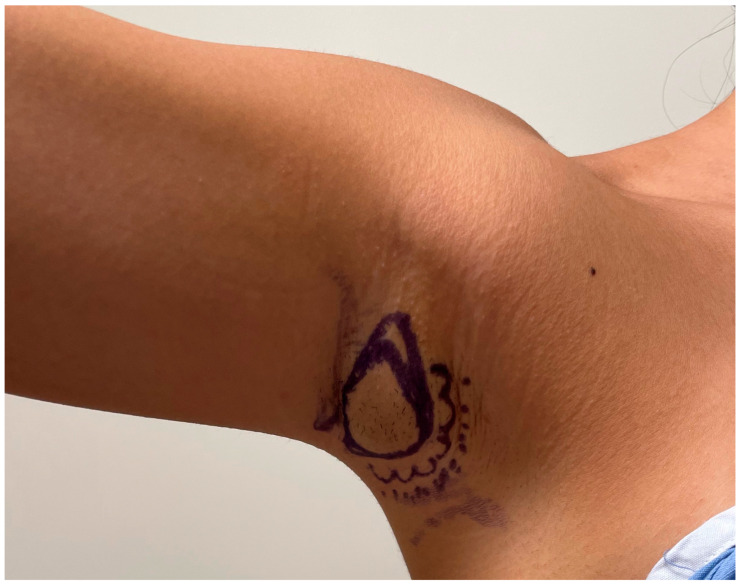
**Preoperative demarcation.** The patient is marked with the arm in 45 degrees abduction. Within the axillary fossa, a crescentic area of excision is marked, including the overlying skin and the underlying adipose and glandular tissue (solid line). The area of dissection that will be beveled outwards is also denoted (cloud line). Any axillary folds or creases, if present, are also demarcated.

Axillary tissue hypertrophy is evaluated with the patient standing and the upper extremities in full abduction, adduction, and various angles in between. The patient is marked with the arm in 45 degrees abduction. Within the axillary fossa, a crescentic area of excision is marked, including the overlying skin and the underlying adipose and glandular tissue. The area of dissection that will be beveled outwards is also denoted. Axillary folds or creases, if present, are also demarcated. The resulting scar is within the axillary fossa so that postoperatively it will not be visible with the patient supine or arm adducted (Figure 2).

### 2.2. Operative Technique

**Figure 3:**
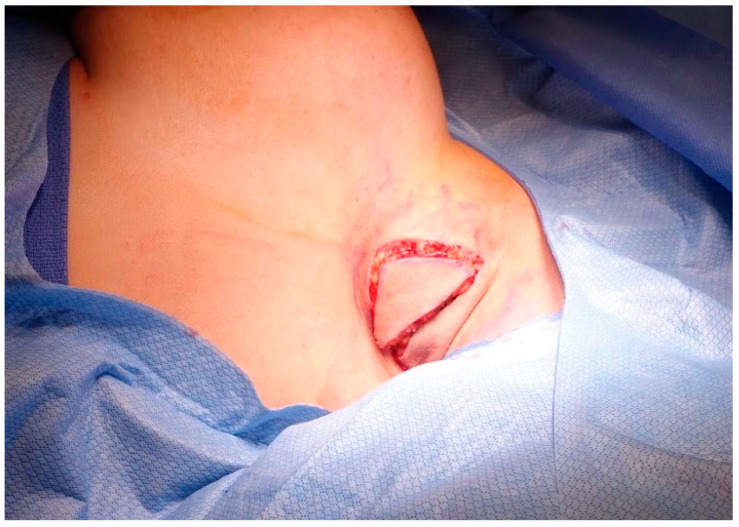
**Axillary tissue incision.** The skin is incised along the marked crescentric area within the axillary fossa. The posterior incision dissection is performed perpendicular, straight down, through the subcutaneous fat.

Prior to surgical incision, antibiotic prophylaxis is administered with the appropriate intravenous antibiotics. The skin is incised and the posterior incision dissection is performed perpendicularly through the subcutaneous fat (Figure 3).

**Figure 4:**
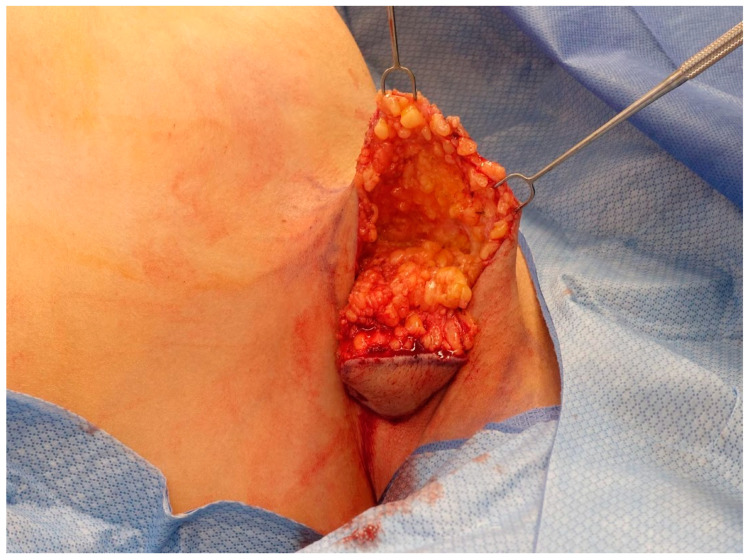
**Tissue beveling and axillary crease obliteration.** After the skin is incised, the dissection is beveled outward anteriorly. This creates a subcutaneous flap anteriorly. At all locations, the dissection is performed down to, but not violating, the clavipectoral fascia. Anteriorly, any axillary creases or folds are obliterated by the scoring of the subcutaneous tissue.

After the skin is incised, the dissection is beveled outward. This creates a subcutaneous flap anteriorly. At all locations, the dissection is performed down to, but not violating, the clavipectoral fascia. Anteriorly, any axillary creases or folds are obliterated by perpendicular scoring of the subcutaneous tissue that creates the fold or crease (Figure 4).

**Figure 5:**
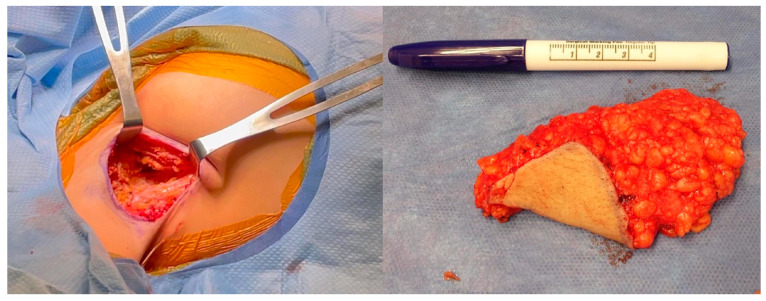
**Axillary tissue excision.** The excised axillary tissue and resulting defect are shown. Removing the area of excess tissue results in dead space that may be prone to serous fluid accumulation. To prevent this, a 15-French Blake drain is placed posteriorly through a separate stab incision.

The axillary ectopic breast tissue is then excised, marked for appropriate laterality, and submitted for permanent pathology (Figure 5).

**Figure 6:**
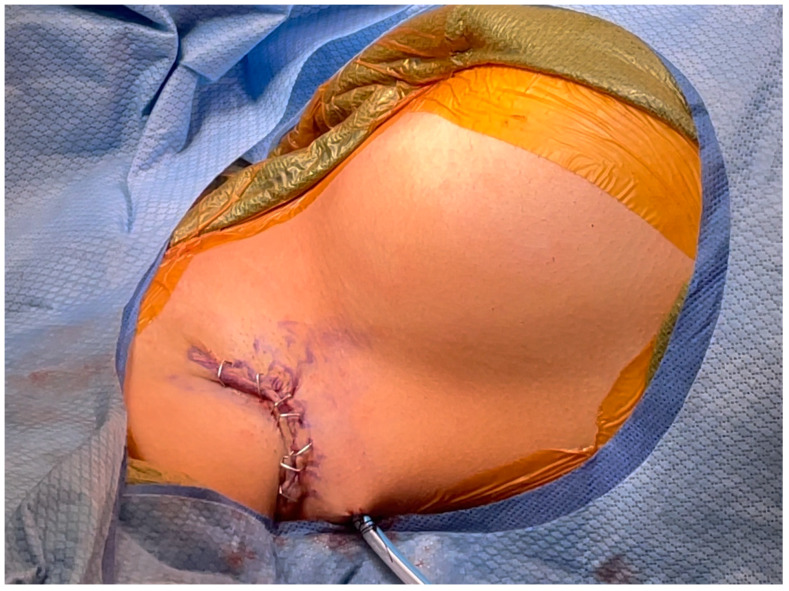
**Meticulous skin alignment for closure.** The skin edges are meticulously aligned at the anterior and posterior edges with redundancy kept in the middle. The incisions are then closed in two layers.

The resulting void was copiously irrigated and hemostasis was ensured. Long-acting anesthetic was used to infiltrate the regional sites for postoperative analgesia. Drain placement varied by surgeon. One author (NT) always placed a 15-French Blake drain posteriorly through a separate stab incision. The other surgeon (BC) only placed a drain in select cases. The skin edges were meticulously aligned at the anterior and posterior edges with redundancy kept in the middle (Figure 6).

The incisions are closed in two layers. An absorbable suture is used in interrupted buried fashion followed by an absorbable subcuticular stitch. 2-octyl cyanoacrylate liquid adhesive and self-adhering mesh (Dermabond Prineo, Ethicon, Raritan, NJ, USA) are placed superficially.

### 2.3. Pathology

Axillary ectopic breast tissue was labeled and fixed in formalin for 64 h, according to the standards set by the American Society of Clinical Oncology (ASCO) and the College of American Pathologists (CAP) to ensure appropriate tissue quality for HER2 receptor testing. The breast specimen ischemic time is 0.0 h, meeting the standard of 1 h or less to ensure appropriate tissue quality for effective estrogen receptor (ER)/progesterone receptor (PR) and HER2 receptor testing. The cut surface is analyzed for tissue identity (breast, adipose, fibrous, and stromal).

### 2.4. Postoperative Management

**Figure 7:**
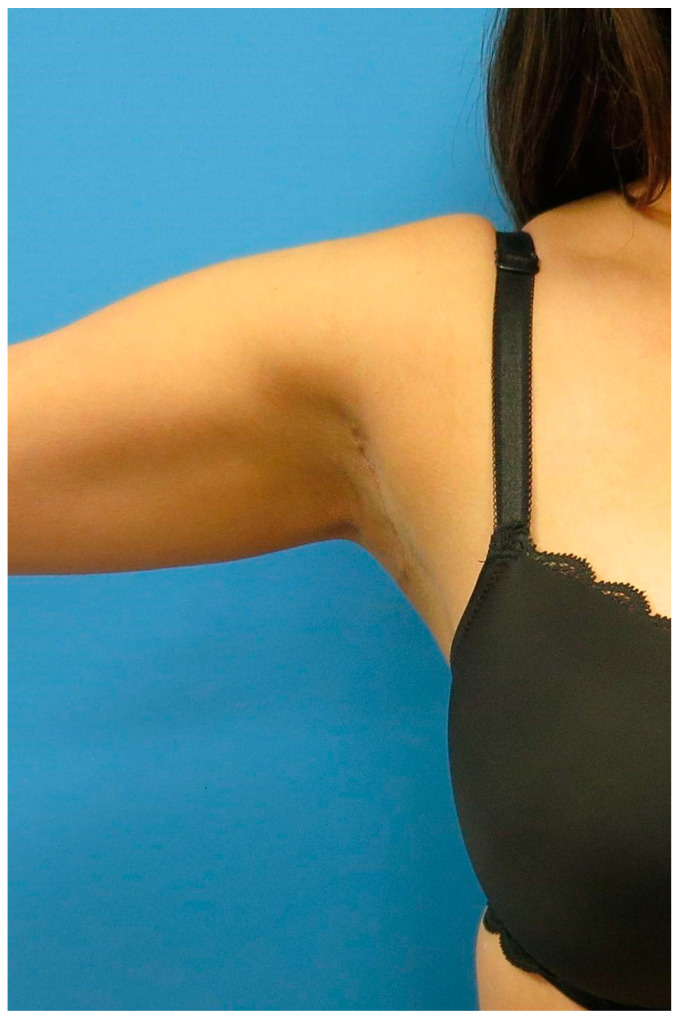
**Postoperative result.** Careful demarcation and incision ensure that the resulting scar is within the axillary fossa. Therefore, postoperatively, the scar will not be visible with the patient supine or arm adducted.

**Figure 8:**
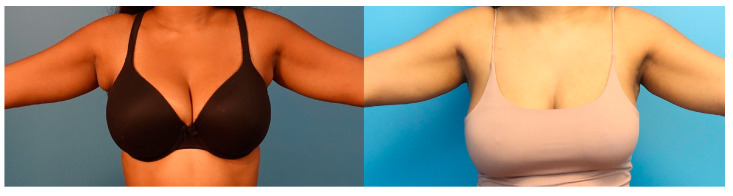
**Before and after.** Preoperative and postoperative comparison of a patient with axillary tissue hypertrophy.

Patients were seen postoperatively at weeks 1, 2, 6, and 12 (Figure 7 and Figure 8).

The drain is removed at the first visit, the adhesive dressing is discontinued at the second visit, and scar care is discussed with the patient at the third visit. Complications warrant more frequent visits.

## 3. Results

### 3.1. Patient Characteristics

During the study period, there were two cohorts of patients treated by two separate surgeons (BC and NT). The BC cohort included 19 women (age range 16 to 59; mean age 41 years), while the NT cohort had 16 women (age range 17 to 53; mean age 36 years). In both cohorts, the majority of patients presented with bilateral axillary ectopic breast tissue (BC 17/19; NT 14/16). All patients complained of aesthetic deformity with significant discomfort. The majority (BC 16/19; NT 13/16) of patients had experienced these symptoms since early adolescence. The remaining three patients began experiencing symptoms during pregnancy. All patients with history of pregnancy noted a worsening of symptoms that did not improve on cessation of breastfeeding. In total, 4 of the 19 patients (21%) in the BC cohort and 7 of the 16 patients in the NT cohort (44%) had concomitant macromastia. The average body mass index (BMI) of all patients in the BC cohort was 26.2 and 27.8 in the NT cohort. More specifically, 4/19 BC patients and 5/16 NT patients were classified as obese with a BMI ≥ 30.

### 3.2. Pathology Reports

All excised axillary tissue was submitted for histopathology. Histologically, all specimens contained benign skin, breast, and adipose tissue. There were no instances of atypia or malignancy.

### 3.3. Complications

Table 1 and Table 2 summarize the complications in both cohorts. Patients had complications requiring some form of intervention, and these included the following.

#### 3.3.1. Cording

Two patients from the NT cohort (12.5%) and one patient from BC cohort (5.2%) presented with axillary cording postoperatively. All three patients were treated with massage and physical therapy, which resulted in complete resolution of the cording and pain. All patients were returned to full range of motion.

#### 3.3.2. Hypertrophic Scarring

Both cohorts (NT 2/16; BC 1/19) had patients with hypertrophic scarring. The quality of the scars included varying degrees of irregularity, widening, and hyperpigmentation. All patients were offered the full spectrum of management options including corticosteroid injections, scar revision, and laser therapy.

#### 3.3.3. Seroma

Seroma rates were higher in the BC cohort (7/19; 37%) as drain placement occurred in only 1/19 patients. Nonetheless, the NT cohort always had drain placement. Despite this universal use of drains, early seroma occurred in 1/16 patients (6.25%). In all patients with postoperative seroma, fluid accumulation occurred early and only a single needle aspiration was utilized to resolve the issue.

**Table 1:** Patient characteristics and outcomes in consecutive order (surgeon = NT).

Pt	Age at Surgery	BMI (kg/m^2^)	Laterality	Complications
1	27	24.8	bilateral (L > R)	None
2	23	21.95	bilateral (L > R)	None
3	23	22.08	bilateral (R > L)	None
4	30	26.3	bilateral (R > L)	None
5	45	29.85	bilateral (L > R)	Mild axillary cording, bilateral. Hypertrophic scar, left
6	49	25.4	bilateral	Seroma, left
7	36	39.44	bilateral	Hypertrophic scar, left
8	32	23.86	bilateral (R > L)	None
9	45	25.82	bilateral (L > R)	None
10	36	27.26	left	Axillary cording, left
11	17	27.46	bilateral (R > L)	None
12	28	35.85	bilateral (R > L)	None
13	52	31.64	bilateral (R > L)	None
14	53	25.61	bilateral (R > L)	None
15	35	31.93	right	None
16	45	25.69	bilateral (R > L)	None

**Table 2:** Patient characteristics and outcomes in consecutive order (surgeon = BC).

Pt	Sex	Age at Surgery	BMI (kg/m^2^)	Laterality	Drain (Y/N)	Complications
**1**	F	43	26.4	right	N	None
**2**	F	51	41.4	bilateral	N	Bilateral axillary seromas
**3**	F	38	18.3	bilateral	N	Bilateral axillary seroma
**4**	F	49	31.32	bilateral	N	Bilateral axillary seroma
**5**	F	54	26.5	bilateral	N	Bilateral axillary seroma
**6**	F	48	27.7	bilateral	Y	None
**7**	F	34	22.7	bilateral	N	Right axillary seroma
**8**	F	44	23	bilateral	N	Bilateral axillary seroma Bilateral cording Small right axillary wound requiring packing/wound care
**9**	F	34	28.97	bilateral	N	None
**10**	F	56	27.2	bilateral	N	None
**11**	F	59	30	bilateral	N	None
**12**	F	16	27.5	bilateral	N	Small Left axillary seroma
**13**	F	26	21	bilateral	N	None
**14**	F	50	26	bilateral	N	None
**15**	F	31	20.8	bilateral	N	None
**16**	F	36	22.3	left	N	None
**17**	F	27	22.1	bilateral	N	Hypertrophic right axillary scar
**18**	F	42	23.6	bilateral	N	None
**19**	F	42	30.8	bilateral	N	None

## 4. Discussion

Axillary tissue hypertrophy is a common condition that has not received much attention in the literature, leading to a lack of consensus on the best treatment for individuals with axillary tissue excess [12]. Axillary ectopic breast tissue can cause pain with cyclic hormonal changes, limitation in activity due to restriction of movement, and sweating or skin irritation [7]. In addition, it can result in cosmetic concerns and aesthetic dissatisfaction. These effects of axillary ectopic breast tissue can cause psychological distress and anxiety for patients; however, the axillary area is often ignored in the evaluation of the aesthetic patient. As such, more attention is needed to optimize the surgical management of these patients.

Various techniques have been proposed in the management of axillary tissue hypertrophy, including surgical excision and liposuction. Although liposuction is less invasive, liposuction can leave behind glandular tissue and/or skin excess that may continue to cause pain or produce suboptimal cosmetic results [13]. On the other hand, surgical excision can result in larger scars and poses an increased risk of complications. A combination of liposuction and surgical excision has been proposed to achieve optimal results, but this case series demonstrates that a modified surgical excision technique alone is a safe and effective method for treating axillary ectopic breast tissue [2].

This study found that the most common complications following accessory tissue removal were axillary cording, hypertrophic scar, and seroma. The existing literature recognizes higher rates of axillary cording, hypertrophic scarring, and seroma after axillary and breast surgery [14,15,16]. Nevertheless, surgeons must establish best practices for managing post-surgical complications. The authors’ recommended management of these complications is as follows:

Cording: While axillary cording (also known as axillary web syndrome (AWS) or lymphatic cording) was found in a small number of patients in this series, the reported incidence of cording after axillary surgery for breast cancer is difficult to ascertain from the current literature. The incidence of cording after axillary lymphadenectomy ranges from 0.6% to 85.4% in the setting of breast cancer, but lower frequencies are reported after sentinel lymph node biopsy (SLNB) compared to axillary lymph node dissection (ALND) [17]. Cording most commonly develops 2–8 weeks after surgery [14]. Given the high incidence of cording following the surgery of the axilla, patients should be counseled of this possibility, and providers must specifically assess for cording at all post-operative visits. Axillary cording can be successfully treated with physical therapy, which includes manual therapy, exercise, and education to improve range of motion and decrease pain [14]. A literature review examining treatment for axillary cording showed that most cases resolved by three months postoperatively with physical therapy, but a small percentage of patients continued to have symptoms of limited shoulder abduction or subjective tightness [18]. In one study with 36 patients with cording, only 3.5% of patients had residual symptoms at twelve weeks [19]. Another study of 56 patients with cording showed 1.7% of patients with residual symptoms at 3 months, and a third study showed resolution in all 44 patients within 2–3 months [20,21].

Hypertrophic Scarring: The medical literature reports a wide range of estimates of the incidence of hypertrophic scarring after axillary surgery. One prospective, multicenter study with 210 patients undergoing ALND showed hypertrophic scarring in 17% of patients [15]. Follow-up in this study included clinical examination of the breast and axilla every three months with a median follow-up time of 29.5 months [15]. Since hypertrophic scarring may require the need for revisionary procedures, patients must be advised about this risk during the informed consent process.

Seroma: Removing the area of excess tissue results in dead space that may be prone to serous fluid accumulation. To prevent this, one author (NT) placed a small drain for a short duration after surgery. Despite this, one patient in the NT cohort presented with seroma after drain removal. As such, the other author (BC) believes that universal drain placement is not necessary. The literature indicates that seroma occurs at rates ranging from 3% to 85% after breast and axillary surgery [16]. The wide range of reported incidence rates in the literature may be due to differing definitions of seroma between papers [16,22].

This series was too small to determine the impact of BMI on the incidence and risk of postoperative complications, but this association is of interest to the authors. The effect of BMI on postoperative complications in axillary ectopic breast tissue resection has not been rigorously studied, but the literature has shown that obesity is linked to an increased risk of surgical complications after reduction mammoplasty. Some of these complications include local surgical site infection, delayed wound healing, wound dehiscence, hematoma, seroma, and tissue necrosis [23]. In contrast, obesity has been studied as a potential protective factor in the development of axillary cording, a finding relevant to this series in which two of the patients developed axillary cording. However, future research is needed to determine if obesity is protective or simply hinders the diagnosis of axillary cording due to excess adipose tissue in the axilla making the palpation of cording more difficult [24].

Limitations of this study include the small sample size of patients, which makes it difficult to analyze patient outcomes due to a lack of statistical power. Additionally, all patients underwent the same procedure for the correction of their axillary tissue hypertrophy. Since there is a lack of an alternative treatment group, it is impossible to compare patient outcomes between different treatment methods in this series. Although this case series demonstrates that surgical excision alone is safe and effective for the treatment of axillary ectopic breast tissue, additional research should evaluate patient-reported satisfaction and compare aesthetic outcomes between different treatment techniques.

## 5. Conclusions

In this study, pathologic evaluation confirmed the successful removal of breast tissue in all patients, indicating that surgical excision is an effective strategy for the removal of ectopic breast tissue. The majority of patients had successful surgeries with minor complications, including seroma, hypertrophic scarring, and axillary cording. This study found that this technique is associated with complications. The most common complications following accessory tissue removal were axillary cording, hypertrophic scars, and seroma.

## Data Availability

The data presented in this study are available on request from the corresponding author. The data are not publicly available due to privacy.
